# A Carrier Phase Ultrafiltration and Backflow Recovery Technique for Purification of Biological Macromolecules

**DOI:** 10.3390/membranes14090188

**Published:** 2024-08-30

**Authors:** Raja Ghosh

**Affiliations:** Department of Chemical Engineering, McMaster University, 1280 Main Street West, Hamilton, ON L8S 4L7, Canada; rghosh@mcmaster.ca; Tel.: +1-905-525-9140 (ext. 27415)

**Keywords:** ultrafiltration, backflow, recovery, purification, proteins, biological macromolecules, sample preparation

## Abstract

A simple carrier phase based ultrafiltration technique that is akin to liquid chromatography and is suitable for medium-to-large volume sample preparation in the laboratory is discussed in this paper. A membrane module was integrated with a liquid chromatography system in a “plug and play” mode for ease of sample handling, and recovery of species retained by the membrane. The sample injector and pump were used for feed injection and for driving ultrafiltration, while the sensors and detectors were used for real-time monitoring of the separation process. The concentration of retained species was enriched by utilizing controlled concentration polarization. The recovery of the retained and enriched species was enhanced by backflow of carrier phase through the membrane using appropriate combination of valves. The backflow of carrier phase also cleaned the membrane and limited the extent of membrane fouling. Proof-of-concept of the proposed technique was provided by conducting different types of protein ultrafiltration experiments. The technique was shown to be suitable for carrying out protein fractionation, desalting, buffer exchange and concentration enrichment. Adoption of this approach is likely to make ultrafiltration easier to use for non-specialized users in biological research laboratories. Other advantages include enhanced product recovery, significant reduction in the number of diavolumes of buffer needed for conducting desalting and buffer exchange, minimal membrane fouling and the potential for repeated use of the same module for multiple separation cycles.

## 1. Introduction

Ultrafiltration is a membrane-based technique that is widely used for the purification of biological macromolecules, such as proteins, carbohydrates and nucleic acids, and ultrafine particles such as viruses, vesicles and virus-like particles (or VLPs) [1,2,3,4,5,6]. It uses membranes that have nanometer-sized pores that can retain substances primarily due to size-based sieving. Other mechanisms such as solute–membrane and solute–solute interactions could also be involved in the retention of a macromolecule by an ultrafiltration membrane [7,8,9,10]. The potential for using ultrafiltration for high-resolution purification of proteins has been demonstrated in several publications [11]. However, the main broad areas of application of ultrafiltration in bioprocesses are concentration, desalting, buffer exchange and ultrafiltration–diafiltration (UFDF) [12,13,14].

Centrifugal ultrafiltration is typically used for carrying out small-volume sample preparation in biological research laboratories [15]. This technique, while being convenient for processing very small volumes of samples, results in low recovery of biological macromolecules [16]. It is also a highly uncontrollable technique with extensive sample handling involved, and therefore, has poor reproducibility. Hence, protocols have to be developed based on guess work and trial and error. Tangential flow filtration (or TFF), which is also sometimes referred to as crossflow filtration, is the technique that is most widely used for large-scale ultrafiltration [17,18]. TFF was originally developed for applications such as the filtration of feed streams containing particulate material such as bacterial and mammalian cells [19,20]. The basic motivation for developing TFF was to avoid build-up of a cake layer on the surface of the membrane, which would otherwise adversely affect the process by reducing the filtration rate and eventually fouling the membrane. In TFF, the feed stream is made to flow parallel to the membrane surface in order to minimize the build-up of retained material. Therefore, only a very small fraction of the fluid entering the membrane module is converted to the filtrate, while the remainder of the feed stream (which is called the retentate) is recycled back to the feed tank. TFF, though widely used for processing biological macromolecules, has several limitations and challenges. To begin with, TFF is a multi-pass process where the biological macromolecules go through the membrane module, pumps and other components of filtration set-up many times. This could result in product degradation through denaturation and uncoiling [21,22]. Problems such as increase in turbidity and aggregation in TFF-processed material have also been widely reported in the literature [23,24,25]. These have been attributed to factors such as high shear rates in the membrane module and in the peristaltic pumps used to generate the tangential flow [23,25,26,27]. Several variants of TFF have been proposed to address some of these limitations [28,29,30].

While TFF and its variants have been successfully used in different bioprocessing applications, these techniques involve dedicated set-ups, each consisting, in addition to the membrane module, of several customized components such as crossflow and permeate pumps, flow sensors, pressure sensors, control valves and a system controller. Therefore, specialized equipment with its associated bench space or floor space allocation is required. While this is not an issue in a bio-manufacturing setting, or indeed in a laboratory that routinely carries out ultrafiltration as its primary activity, this could be a limiting factor in most biological research laboratories that mainly use ultrafiltration for applications such as small-to-medium volume sample preparation or for biological product development and testing. Some of these laboratories typically lack the required infrastructure and expertise for operating and maintaining dedicated TFF systems, and therefore, rely primarily on centrifugal ultrafiltration, which is limiting in terms of its low speed, capacity, controllability, reproducibility and product recovery. Also, centrifugal ultrafilters are single-use devices that need to be discarded after each sample processing.

In this paper, a simple ultrafiltration technique that is suitable for processing larger sample volumes than that possible using centrifugal ultrafiltration is discussed. In addition, it addresses some of the challenges associated with the incumbent laboratory techniques mentioned in the previous paragraph, i.e., low speed, low controllability and low product recovery. The proposed technique, which is a modified version of the stirred-cell filtration-based carrier phase ultrafiltration technique discussed earlier [21,31], is carried out by integrating a membrane module with a liquid chromatography system using appropriate tubing, valves and connectors. A carrier phase, which is analogous to the mobile phase used in liquid chromatography is a buffer that is appropriate for a given separation. The proposed technique utilizes the pump and the sample injector of the liquid chromatography system for handling the feed solutions. The pump is also used to drive the ultrafiltration process. The UV and pH detectors and the pressure sensor of the liquid chromatography system are used for real-time process monitoring. Thus, the operation of the proposed ultrafiltration technique is akin to liquid chromatography, i.e., the ultrafiltration process could be carried out in a “plug and play” mode by integrating the membrane module, connectors and valves, as and when required, with a liquid chromatography system, which is typically a standard fixture in most biological research laboratories. This technique is, therefore, likely to make ultrafiltration easier to use for the non-specialized user. In addition, there are other benefits, as discussed in this paper, such as enhanced recovery, real-time monitoring of parameters such as biomacromolecule concentration and conductivity, reduction in the number of diavolumes of buffer needed for desalting and buffer exchange, the possibility to conduct multiple separation cycles using the same module and the potential for carrying out biomacromolecule–biomacromolecule fractionation. 

Ultrafiltration membrane modules used for TFF, and its variants are typically designed to accommodate large flow rates on the feed side of the membrane since the high shear rate resulting from such crossflow is vital for efficient operation. Therefore, if such modules were to be used for applications such as small- and medium-scale sample processing and preparation, the achievable concentration enrichment factor would be limited, i.e., the retained species samples obtained would not be very concentrated. Therefore, for such applications, a membrane module with very low hold-up of liquid would be desirable. Laterally fed membrane devices that have been proposed for chromatographic separations are designed such that the hold-up volume is minimized [32,33]. These devices are also designed with customized flow channels for efficient flow distribution and collection [32,33]. Such flow features and attributes are also desirable in the ultrafiltration technique proposed in this paper. Therefore, a repurposed laterally fed membrane device was used in the current study to conduct the ultrafiltration experiments. Any standard membrane module designed for TFF-type application could also have been used for this technique. However, it could be anticipated that the efficiency of separation obtained with such uncustomized modules would be lower. 

**Figure 1:**
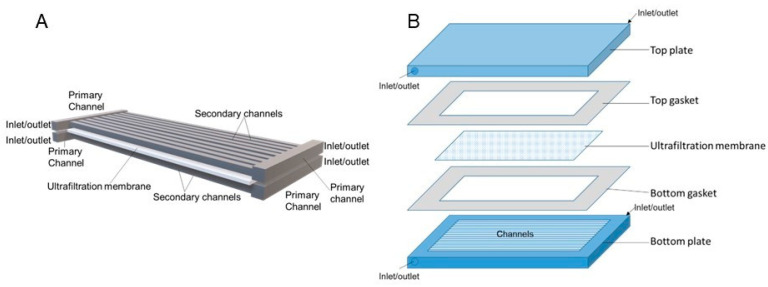
(**A**) Flow channels, inlets/outlets and position of membrane in a laterally fed ultrafiltration device. (**B**) The different components used to assemble a laterally fed ultrafiltration device for the carrier phase ultrafiltration and backflow recovery technique.

**Figure 2:**
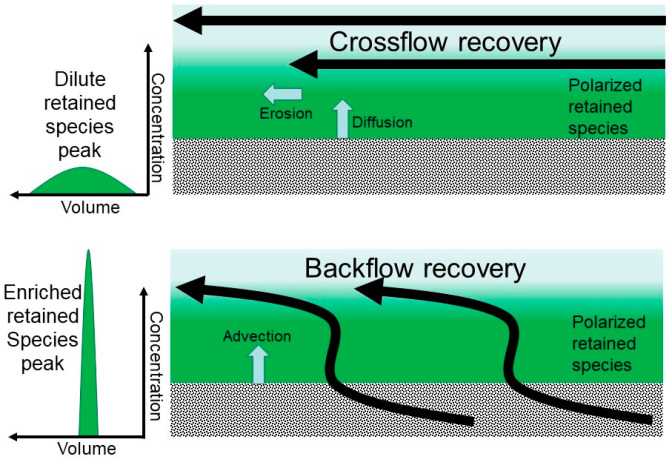
Comparison of the mechanisms involved in the recovery of retained species by crossflow and backflow.

The flow distribution and collection plates of a z^2^ laterally fed membrane chromatography device [34] were repurposed for use in an ultrafiltration process. Figure 1A shows the flow distribution and collection channels, inlet/outlet ports and the location of the ultrafiltration membrane in the module used in the proposed technique. Figure 1B shows how the plates and the ultrafiltration membrane are assembled and sealed together as a module using appropriate gaskets. During the filtration steps of the proposed technique, the fluid (i.e., the feed solutions or the carrier phase) is introduced on the feed side of the ultrafiltration membrane using one of the two ports on that side of the module. The fluid is distributed to the secondary channels where it initially flows parallel to the membrane surface and eventually through the membrane as the other port on the feed side is closed. Therefore, the manner in which the fluid is handled in the proposed technique, while being similar to that in dead-end filtration, also has some attributes of crossflow filtration. After the permeable species have been completely (or satisfactorily) removed in the filtrate stream, the species retained by the membrane within the membrane module is recovered. Recovery could be achieved in the conventional crossflow mode by opening the second port on the feed side and flushing out the retained species using a carrier phase (see Figure 2). A more efficient way to recover the retained species that is utilized in the proposed technique is by using backflow of carrier phase through the membrane (see Figure 2). The use of backflow or backwash or backpulsing or alternating permeate flow direction for reducing fouling of ultrafiltration membrane and for restoring permeate flux has been reported in the literature [35,36,37,38]. The use of backwash to enhance recovery of viruses present in water samples during filtration with hollow fiber membranes has also been demonstrated [39,40]. As mentioned earlier, significant accumulation of retained species takes place very close to the surface of an ultrafiltration membrane due to a phenomenon called concentration polarization [41]. Indeed, in the proposed technique, controlled concentration polarization is actually utilized for concentration enrichment of the retained species. Therefore, backflow would not only result in more complete recovery and of the retained species but would also clean the membrane surface, thereby limiting the extent of membrane fouling, and make the membrane module ready for the next separation cycle.

In this study, lysozyme and BSA were used as model permeable and retained biological macromolecules, respectively. The filtration step of the ultrafiltration technique was standardized using a lysozyme feed solution, while the recovery step was standardized using a BSA feed solution. After this, the feasibility of fractionating lysozyme and BSA using the proposed ultrafiltration technique was examined. Experiments were then carried out using BSA solution containing high salt concentration to demonstrate that the technique was suitable for desalting and buffer exchange of biological macromolecules. Finally, experiments were carried out using dilute and moderately concentrated BSA feed solutions to demonstrate that the proposed ultrafiltration and backflow recovery technique was suitable for enriching the concentration of biological macromolecules. The use of volumetric permeate flux manipulation for controlling the enrichment factor in such concentration processes was also demonstrated. In these experiments, the pressure drop was recorded to assess the extent of membrane fouling. The results obtained are discussed. 

## 2. Materials and Methods

Bovine serum albumin (A9418), lysozyme (L6876), sodium phosphate dibasic (S0876), potassium chloride (P9541), potassium phosphate monobasic (P5655) and sodium chloride (S7653) were purchased from Sigma-Aldrich (St. Louis, MO, USA). Ultrafiltration membrane (30 kDa MWCO, polyethersulfone, OMEGA 30K Part# OT30SHEET) was purchased from Pall Corporation Canada, Mississauga, ON, Canada. All buffers and proteins solutions were prepared using water (18.2 MΩ cm) obtained from a SIMPLICITY 185 water purification unit (Millipore, Molsheim, France). Buffers used as carrier phase in the ultrafiltration experiments were degassed and microfiltered using 0.1 µm pore size microfiltration membrane prior to their use in the ultrafiltration experiments.

The distribution and collection plates (see Figure 1B) used to assemble the ultrafiltration module were made of acrylic. The design of these plates was based on those used for z^2^ laterally fed membrane chromatography and is discussed in detail elsewhere [34]. Briefly, the plates were provided with embedded primary channels, which were drilled within these acrylic plates. The secondary channels on these plates were created by machining, and these were in turn connected to their corresponding primary channels through small connecting holes [34]. The effective area of the ultrafiltration membrane, which corresponded to that covered by the secondary channels on a plate, was 55.5 cm^2^ (4.7 cm × 11.8 cm). A rectangular piece of ultrafiltration membrane was sandwiched and sealed between the top and bottom plates, as shown in Figure 1B, using rubber gaskets.

**Figure 3:**
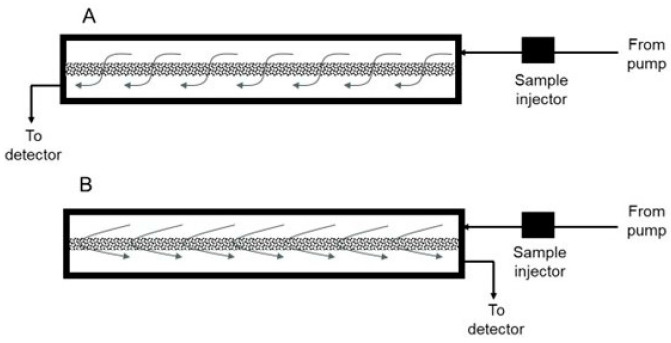
Set-ups used for monitoring the clearance of permeable species from the membrane module by S-flow ultrafiltration (**A**) and C-flow ultrafiltration (**B**).

**Figure 4:**
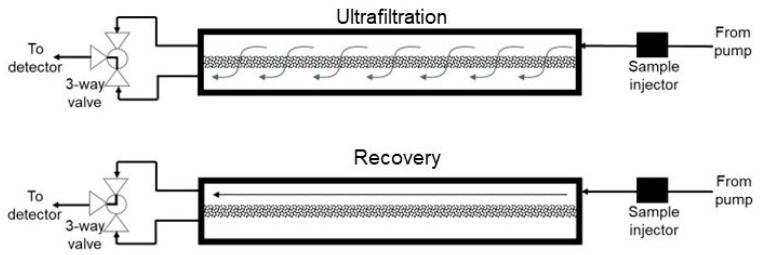
Set-up used for S-flow ultrafiltration crossflow recovery.

**Figure 5:**
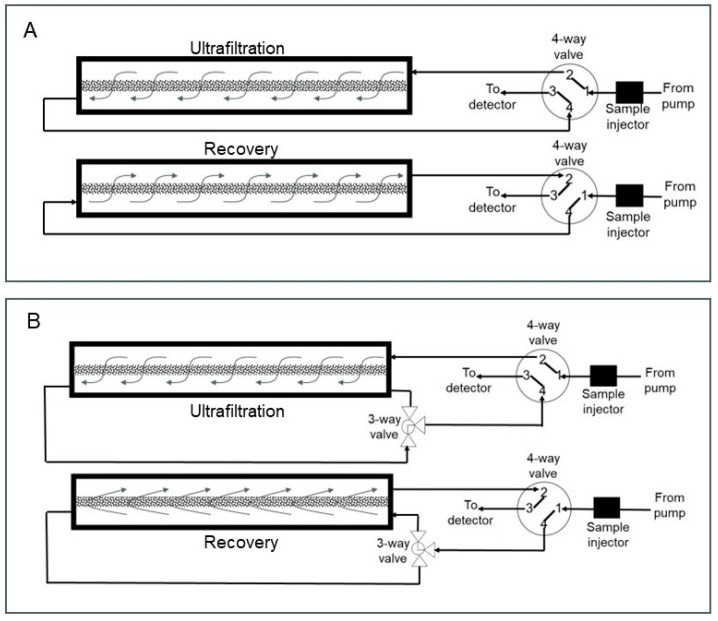
Set-ups used for S-flow ultrafiltration S-backflow recovery (**A**) and S-flow ultrafiltration C-backflow recovery (**B**).

**Figure 6:**
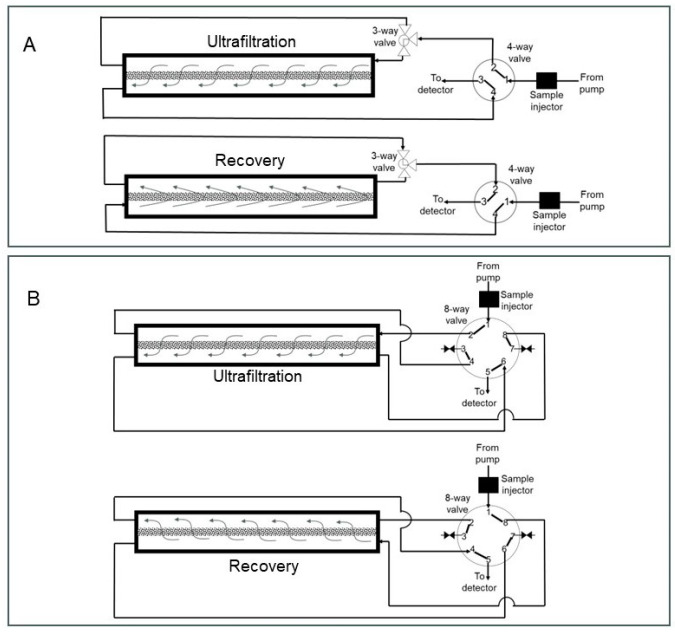
Set-ups used for S-flow ultrafiltration flipped C-backflow recovery (**A**) and S-flow ultrafiltration flipped S-backflow recovery (**B**).

The assembled z^2^ laterally fed ultrafiltration module was integrated with an AKTA Prime Plus liquid chromatography system (GE Healthcare Biosciences, Montreal, QC, Canada) using appropriate PEEK tubing, connectors and valves. Phosphate-buffered saline, pH 7.4 (or PBS), was used as the carrier phase, while lysozyme and BSA, respectively, were used as the model permeable protein and retained protein. Several different set-ups were examined for the proposed carrier phase ultrafiltration and backflow recovery technique, and these are described individually. The effect of the type of flow within the z^2^ laterally fed ultrafiltration module on the clearance of permeable species through the ultrafiltration membrane was examined using two different set-ups (see Figure 3). In the first of these (see Figure 3A), the feed inlet and filtrate outlet were located at diagonally opposite ends. The resultant flow during ultrafiltration is termed S-flow ultrafiltration due to the overall direction of fluid flow within the membrane module. In the second set-up used for testing clearance of permeable species (see Figure 3B), the feed inlet and filtrate outlet were located on the same end of the module. Due to its overall direction within the membrane module, the resultant fluid flow is termed C-flow ultrafiltration. The effect of overall directions of fluid flow within the membrane module on the recovery of species retained by the ultrafiltration membrane was examined using the five different set-ups shown in Figure 4, Figure 5 and Figure 6. Figure 4 shows the set-up used for S-flow ultrafiltration crossflow recovery. Here, the ultrafiltration step of the process was carried out using S-flow while the recovery step was carried out in the crossflow mode by switching the position of a three-way valve, as shown in the figure. Figure 5A shows the set-up used for carrying out S-flow ultrafiltration S-backflow recovery, which was carried out by switching the position of a four-way valve, as shown in the figure. Figure 5B shows the set-up used for carrying out S-flow ultrafiltration C-backflow recovery using a combination of one three-way and one four-way valve. Figure 6A shows the set-up used for carrying out S-flow ultrafiltration flipped C-backflow recovery. This set-up also consisted of a combination of one three-way valve and one four-way valve, but the location of the three-way valve was different from that used for S-flow ultrafiltration C-backflow recovery. Figure 6B shows the set-up used for carrying out S-flow ultrafiltration flipped S-backflow recovery using an eight-way valve. 

## 3. Results and Discussion

**Figure 7:**
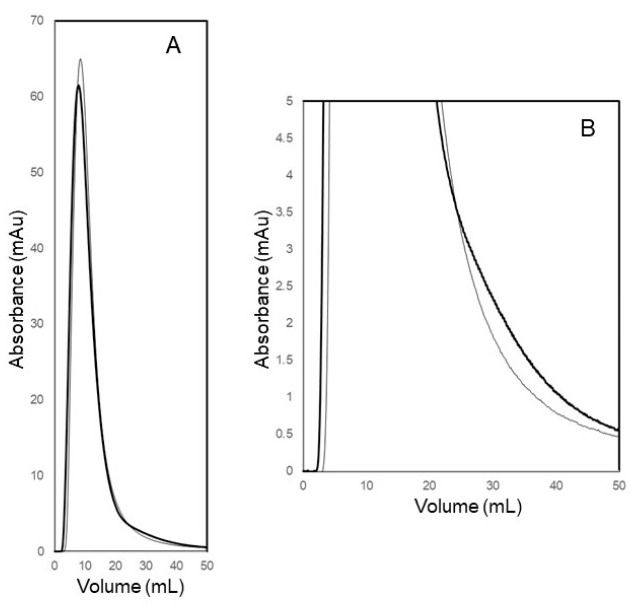
(**A**) UV absorbance profiles of permeate obtained during the clearance of lysozyme from the membrane module using S-flow ultrafiltration (thin line) and C-flow ultrafiltration (thick line). (**B**) Close up on the start and tail sections of the lysozyme clearance profiles.

Figure 7 shows the UV absorbance profiles of the filtrate obtained during experiments carried out to study the effect of the overall filtrate flow direction, i.e., S-flow or C-flow ultrafiltration on the clearance of a permeable biological macromolecule through an ultrafiltration membrane. In these experiments, lysozyme (0.25 mg mL^−1^ solution prepared in PBS) was used as the model permeable species, and PBS was used as the carrier phase. These experiments were carried out at a filtration flow rate of 5 mL min^−1^, which corresponded to a volumetric permeate flux of 1.5 × 10^−5^ m s^−1^ (or 54.1 L m^−2^ h^−1^). Each experiment was started by filtering the carrier phase through the ultrafiltration membrane until stable pressure and UV absorbance baselines were obtained. Then, 5 mL of lysozyme solution was injected into the membrane module using a sample injector. The UV absorbance profile of the permeate obtained in each experiment was analyzed to determine the efficiency with which lysozyme was cleared from the membrane module. From a comparison of the profiles in Figure 7, it may be inferred that S-flow ultrafiltration resulted in faster clearance of lysozyme through the 30 kDa MWCO membrane. Due to this, S-flow ultrafiltration was selected for all subsequent experiments.

**Figure 8:**
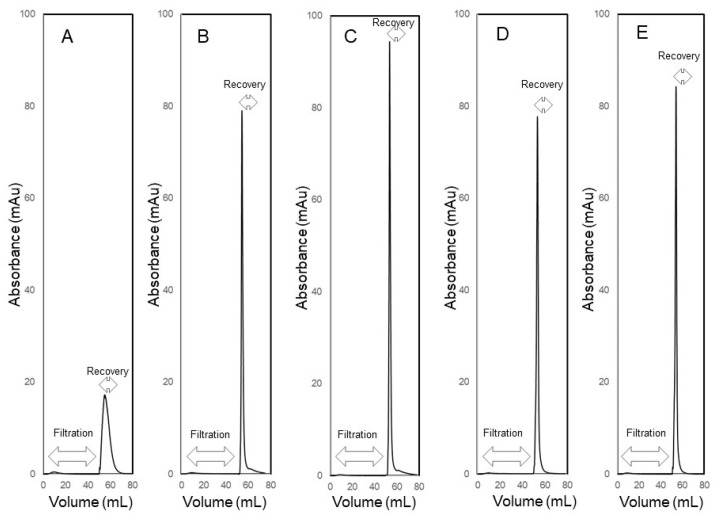
UV absorbance profiles obtained during recovery of BSA using S-flow ultrafiltration crossflow recovery (**A**), S-flow ultrafiltration S-backflow recovery (**B**), S-flow ultrafiltration C-backflow recovery (**C**), S-flow ultrafiltration flipped C-backflow recovery (**D**) and S-flow ultrafiltration flipped S-flow recovery (**E**).

Figure 8 shows the UV absorbance profiles obtained during the filtration and recovery steps of experiments carried out to examine the effect of the mode of recovery, i.e., crossflow and the different types of backflows, on the efficiency of recovery of a retained biological macromolecule (BSA) from the membrane module. These experiments were carried out by injecting 5 mL of BSA feed solution (0.25 mg mL^−1^ prepared in PBS). The filtration/recovery flow rate was 5 mL min^−1^, which corresponded in the case of the filtration step to a volumetric permeate flux of 1.5 × 10^−5^ m s^−1^ (or 54.1 L m^−2^ h^−1^). In each experiment, 45 mL of carrier phase was filtered through the membrane module after the feed injection. The UV absorbance of the permeate remained very close to the baseline, indicating that BSA was almost totally retained by the 30 kDa MWCO ultrafiltration membrane. With crossflow recovery (Figure 8A), BSA was obtained as a broad peak, i.e., as a dilute sample, which indicated that this mode of recovery was not very efficient. By contrast, during backflow recovery (see Figure 8B–E), the retained BSA was obtained as concentration-enriched samples as evident from the sharp recovery peaks. These results clearly indicate that backflow was a significantly more efficient way than crossflow for recovering retained species from the ultrafiltration module. During crossflow recovery, the BSA molecules that were enriched near the membrane surface due to concentration polarization were transported to the bulk carrier phase primarily by the scouring action of flowing fluid, i.e., by erosion of the concentration polarization layer, and to some extent by diffusion (as hypothesized in Figure 3A). Therefore, there was significant dilution of the retained BSA during crossflow recovery. On the other hand, during backflow recovery, the retained BSA was more efficiently recovered by the carrier phase by advection (as hypothesized in Figure 3B), without the dilution effect observed with crossflow. There were some differences observed with the different types of backflows, i.e., some of the recovery peaks were sharper than others. In chromatography, an objective way to compare sharpness of peaks is based on their widths at half height. This approach was borrowed here to compare the BSA recovery peaks and thereby enable a quantitative result-based decision about which type of backflow was most efficient. The BSA peak widths obtained during crossflow and the different types of backflow recovery are summarized in Table 1. These results firstly show that the BSA peak obtained during crossflow recovery was significantly wider than those obtained during all the different types of backflow recovery. Among these different types, C-backflow recovery resulted in the sharpest BSA peak. Based on this, all subsequent experiments in this study were carried out by S-flow ultrafiltration C-backflow recovery.

The sharp BSA peaks obtained during backflow recovery clearly indicate that the retained BSA was enriched within a small zone within the membrane module, which could only be the concentration polarization layer. The sharp peaks also confirm the significant role played by advection during backflow of carrier phase in maintaining this concentrated band and thereby ensuring efficient recovery of BSA. As mentioned earlier, the dynamic and reversible accumulation of retained species close to the membrane surface is referred to as concentration polarization [41,42]. In conventional filtration processes such as TFF and dead-end filtration, where the feed solution is pumped into the membrane module in a sustained manner, prolonged concentration polarization on the membrane has been linked to problems such as membrane fouling, i.e., attachment or deposition of retained species on the membrane [42,43]. Membrane fouling leads to the degradation of membrane performance and ultimately necessitates either cleaning or replacement of the membrane [43]. In steady-state ultrafiltration, where there is sustained flow of feed into the membrane module, the concentration of the retained species adjacent to the membrane surface (*C_w_*) can be determined using the modified form of the concentration polarization equation [21]:

*C_w_* = *C_b_* exp ((*J_v_ δ*)/*D*) (1)

where *C_b_* is the concentration of the species in the bulk feed, *J_v_* is the volumetric permeate flux, *δ* is the thickness of the concentration polarization layer and *D* is the diffusivity of the retained species. In the proposed carrier phase ultrafiltration and backflow recovery technique, where a fixed amount of feed solution is pulsed into the membrane module, as *C_w_* increases due to accumulation of the retained species, the value of *C_b_* decreases due to depletion from the bulk feed. Therefore, there is an iterative adjustment of the two concentrations before a steady-state concentration profile is formed within the concentration polarization layer. Therefore, the extent of concentration polarization in the proposed technique would be limited by the amount of sample injected and, hence, be controllable. Indeed, such controlled concentration polarization could be utilized to enrich the retained species in a controllable manner by adjusting the volumetric permeate flux (as is discussed later in this paper). Also, such controlled enrichment of retained species by concentration polarization is carried out for a relatively short duration and is immediately followed by the removal of the concentration polarization layer by backflow of carrier phase through the membrane during recovery. Therefore, the backflow serves three purposes, i.e., the recovery of enriched retained species without significant dilution, elimination of concentration polarization and cleaning of the membrane surface. The extent of membrane fouling is, therefore, expected to be low in the proposed carrier phase ultrafiltration and backflow recovery technique.

**Figure 9:**
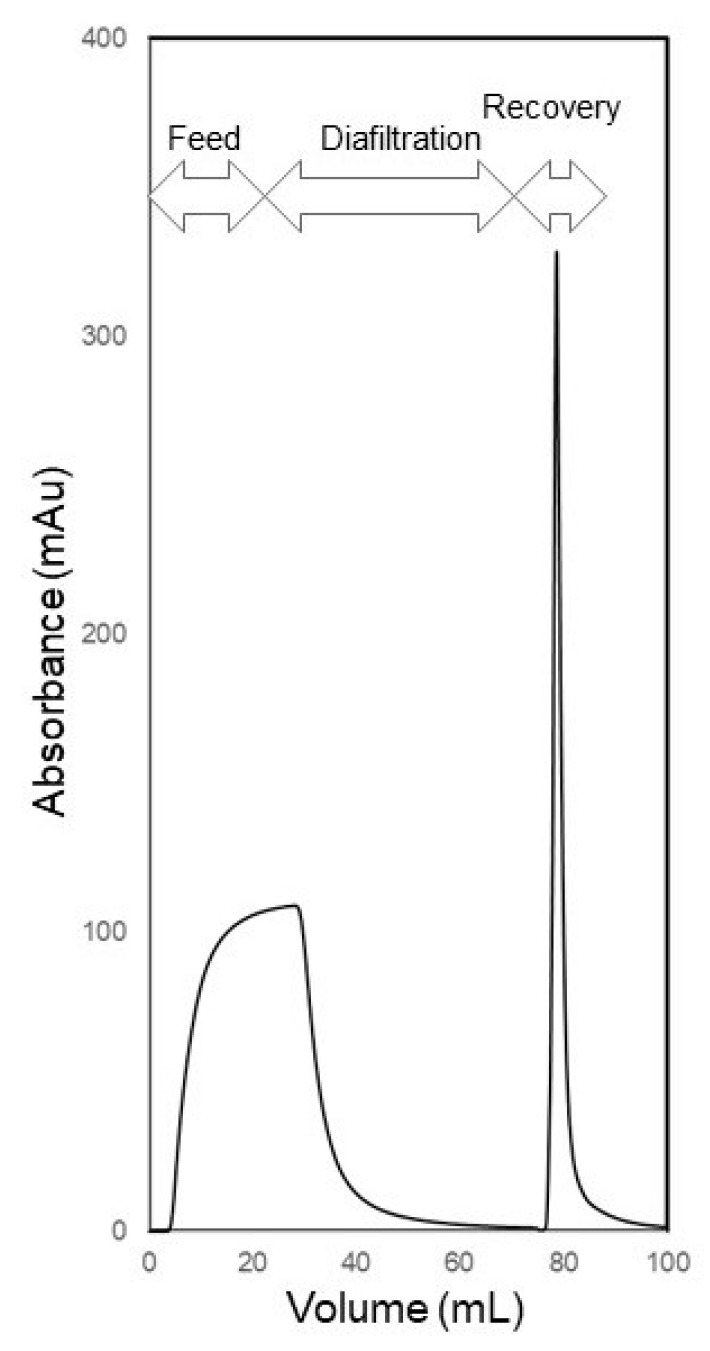
UV absorbance profile obtained during the fractionation of lysozyme (permeable protein) and BSA (retained protein) by S-flow ultrafiltration C-backflow recovery.

Figure 9 shows the UV absorbance profile obtained during fractionation of lysozyme (the permeable species) and BSA (the retained species) using the carrier phase ultrafiltration and backflow recovery technique. This experiment was operated in the S-flow ultrafiltration C-backflow recovery mode using a feed solution consisting of 0.25 mg mL^−1^ lysozyme and 0.25 mg mL^−1^ BSA prepared in PBS. The flow rate used during the filtration and recovery steps of the separation process was 5 mL min^−1^ (volumetric permeate flux during filtration = 1.5 × 10^−5^ m s^−1^ (or 54.1 L m^−2^ h^−1^). The filtration step in this experiment consisted of two sub-steps: feed injection and diafiltration. The volume of feed solution injected was 25 mL, and this was chased using 50 mL of carrier phase during diafiltration. By the end of the diafiltration sub-step, the UV absorbance of the permeate reached the baseline, indicating that lysozyme had been completely cleared form the membrane module. The BSA retained and enriched by controlled concentration polarization within the membrane module was then recovered and obtained in the form of a sharp peak. These results demonstrate that the proposed technique could be used for the efficient fractionation of retained and unretained biological macromolecules.

**Figure 10:**
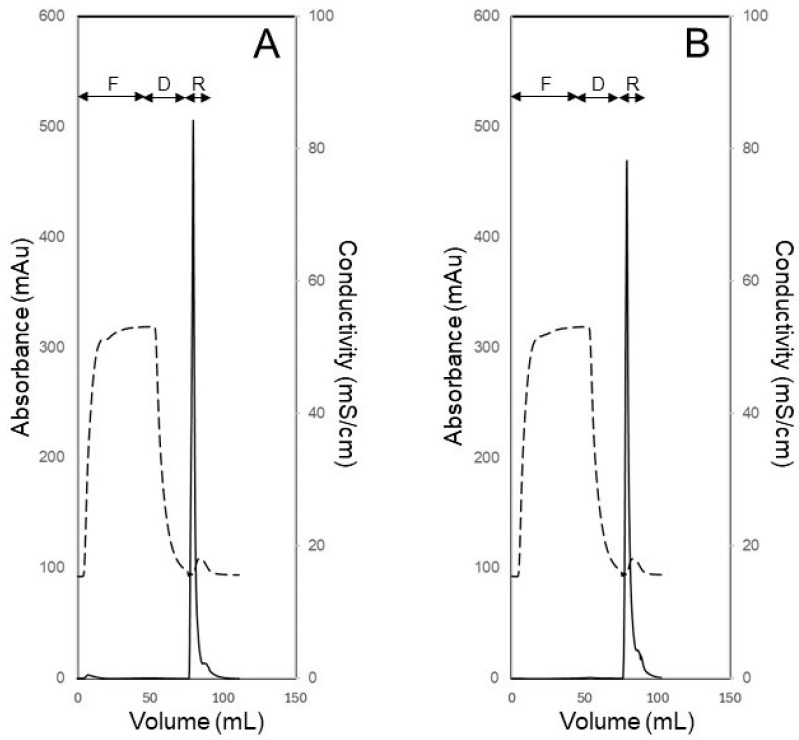
Desalting of 0.25 mg mL^−1^ BSA solution (50 mL feed volume) by S-flow ultrafiltration C-backflow recovery at 4 mL min^−1^ flow rate (**A**) and at 5 mL min^−1^ flow rate (**B**) (F: feed injection, D: diafiltration, R: recovery, solid line: UV absorbance and dashed line: conductivity).

Figure 10 shows the UV absorbance and conductivity profiles obtained during carrier phase ultrafiltration and backflow recovery experiments operated in the S-flow ultrafiltration C-backflow recovery mode for desalting a solution of BSA. These experiments were carried out with feed solution consisting of 0.25 mg mL^−1^ BSA prepared in PBS and further containing 1 M sodium chloride. The objective of these experiments was to use PBS as carrier phase to remove excess sodium chloride from the BSA sample. Figure 10A,B show the UV absorbance and conductivity profiles obtained from experiments carried out at 4 mL min^−1^ and 5 mL min^−1^ flow rates, respectively. In each experiment, 50 mL of BSA solution was first injected into the membrane module. This was followed by diafiltration using 25 mL of carrier phase. The progress of the desalting process was monitored by tracking the conductivity of the filtrate, and an informed decision based on real-time information about when to stop the diafiltration sub-step and switch to the recovery step was made. The desalted and concentrated BSA sample was recovered by backflow of carrier phase in the form of sharp peaks. In each experiment, the conductivity decreased from about 53 mS cm^−1^ (the 1 M NaCl containing BSA feed sample) to about 16 mS cm^−1^ (the recovered BSA sample), which indicated that sodium chloride was removed quite effectively. The above desalting protocol could also be used for buffer exchange of solutions of biological macromolecules. The time required in these experiments for desalting 50 mL of protein solution were 20 min and 25 min, respectively, at 5 mL min^−1^ and 4 mL min^−1^ flow rates, which were quite fast considering the relatively large volume of sample processed (compared to centrifugal ultrafiltration). Also, in centrifugal ultrafiltration, several consecutive spins are required for satisfactory desalting. The buffer consumption in desalting and buffer exchange process is typically measured in terms of the number of required diavolumes, which is defined as the volume of buffer needed divided by the feed volume [44]. The typical value for the number of diavolumes of buffer required for satisfactory desalting or buffer exchange is about five or greater [44]. In the experiments represented in Figure 10, the volume of diafiltration buffer (carrier phase) used to desalt the BSA solution was just half of that of the feed volume, i.e., the number of diavolumes of buffer used was 0.5. Therefore, adoption of the proposed technique would result in significant reduction in buffer consumption during desalting and buffer exchange processes. An interesting observation based on the comparison of the BSA recovery peaks shown in Figure 10A,B is that desalting at a flow rate of 5 mL min^−1^ resulted in a slightly greater enrichment in recovered protein concentration. This effect of flow rate (and hence volumetric permeate flux) on recovered protein concentration is examined in detail in the next paragraph in the context of BSA concentration enrichment experiments.

**Figure 11:**
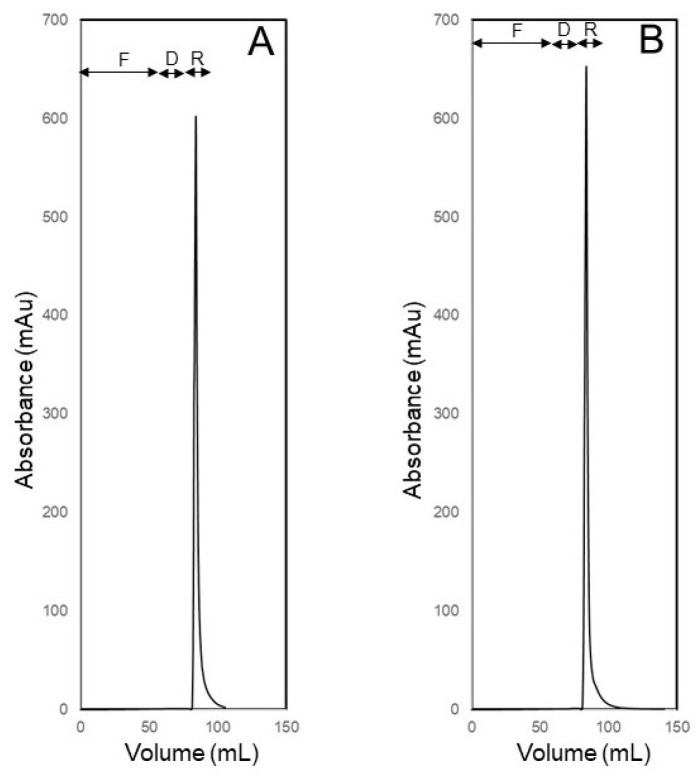
Concentration of 0.25 mg mL^−1^ BSA solution (70 mL feed volume) by S-flow ultrafiltration C-backflow recovery using 4 mL min^−1^ flow rate during both feed injection and diafiltration (**A**) and using a combination of 4 mL min^−1^ flow rate during feed injection and 5 mL min^−1^ flow rate during diafiltration and recovery (**B**) (F: feed injection, D: diafiltration and R: recovery).

Figure 11 shows the UV absorbance profiles obtained during BSA concentration experiments carried out in the S-flow ultrafiltration C-backflow recovery mode. The BSA concentration in the feed solution prepared using PBS was 0.25 mg mL^−1^, and 70 mL of this was injected into the membrane module in these experiments. The feed injection sub-step in both experiments were carried out at 4 mL min^−1^ flow rate. In each experiment, the feed injection sub-step was followed by diafiltration with 10 mL of carrier phase, the flow rates during diafiltration being different, i.e., 4 mL min^−1^ in one experiment (see Figure 11A) and 5 mL min^−1^ in the other (see Figure 11B). Here, the objective was to see if the increase in concentration polarization at the higher volumetric permeate flux during diafiltration had any impact on the concentration of the recovered BSA sample. After these respective diafiltration steps, the retained BSA was recovered by C-backflow. By comparing the recovered BSA peaks shown in Figure 11A,B, it may be inferred that greater concentration enrichment occurred when the flow rate was bumped up during diafiltration and recovery. To verify this, the protein concentrations in the BSA samples recovered during these experiments were measured and compared with that in the original feed solution. The concentration enrichment factors obtained using diafiltration flow rates of 4 mL min^−1^ and 5 mL min^−1^ were 10.5 and 11.8, respectively, while the corresponding BSA recovery values in these samples were 97.1% and 99.6%, respectively. These recoveries were significantly greater than the typical recoveries reported in the literature for centrifugal ultrafiltration [15,45]. These results demonstrate that the carrier phase ultrafiltration and backflow recovery technique is suitable for concentrating dilute solutions of biological macromolecules by more than an order of magnitude. Also, the concentration enrichment factor could be changed by manipulating the extent of concentration polarization by appropriately adjusting the volumetric permeate flux through the retaining ultrafiltration membrane. The high recovery of BSA also suggests that the extent of membrane fouling in these experiments was very low as very negligible amounts of foulant (i.e., BSA) were left behind on the ultrafiltration membrane.

**Figure 12:**
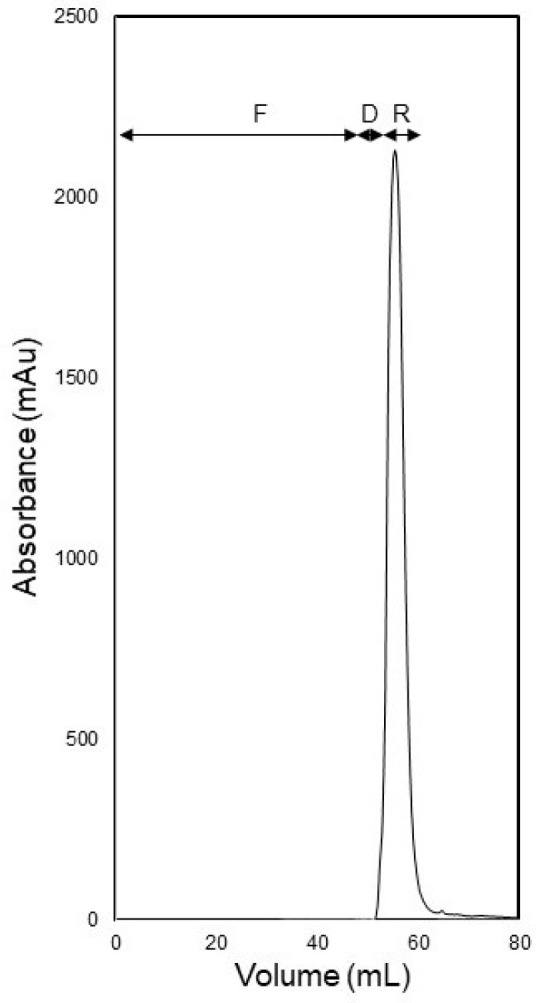
Concentration of 2 mg mL^−1^ BSA solution (50 mL feed volume) by S-flow ultrafiltration C-backflow recovery using 1 mL min^−1^ flow rate during feed injection and 2 mL min^−1^ flow rate during diafiltration and recovery (F: feed injection, D: diafiltration and R: recovery).

Figure 12 shows the UV absorbance profile obtained during concentration enrichment of a BSA solution having a concentration of 2 mg mL^−1^ prepared in PBS. In this experiment, 50 mL of feed solution was injected into the membrane module at a flow rate of 1 mL min^−1^. The higher concentration of the BSA feed solution (eight times higher than that in the concentration enrichment experiments discussed in the previous paragraph) necessitates the use of a lower feed flow rate. The ultrafiltration sub-step was followed by diafiltration using 7.5 mL of carrier phase. During diafiltration, the flow rate was bumped up to 2 mL min^−1^ to enrich the retained protein further by concentration polarization. The retained and concentrated BSA sample was then recovered from the membrane module by C-backflow and was obtained in the form of a sharp peak. The protein concentration of the recovered BSA sample was measured to determine concentration enrichment factor and recovery. These were found to be 6.7 and 97.2%, respectively. These results show that the proposed carried phase ultrafiltration and backflow recovery technique was also suitable for processing moderately concentrated solutions of biological macromolecules.

Membrane fouling is sometimes a limiting factor in ultrafiltration. The proposed technique relies heavily on concentration polarization for enrichment of retained species. While concentration polarization and fouling are distinctly different [42,43], prolonged concentration polarization could promote membrane fouling. Also, fouling would be more severe if the process were operated above the critical flux for the system [42]. The extent of membrane fouling in the proposed technique was assessed by comparing the overall pressure data recorded by the liquid chromatography system during filtration of carrier phase before and after carrying out a particular protein ultrafiltration experiment. The overall pressure included both the system pressure and the transmembrane pressure. However, it was found that the transmembrane pressure was the major contributor to the overall pressure. Moreover, the system pressure was independent of what happened within the membrane module. Hence, the overall pressure was a satisfactory metric for quantifying membrane fouling, i.e., a higher pressure during carrier phase filtration after a protein separation experiment compared to that before it would indicate fouling. Table 2 summarizes the overall pressure observed during filtration of carrier phase both before and after a particular type of protein separation experiment. These results suggest that there was minimal amount of fouling of the ultrafiltration membrane used in this study. 

The main purpose of the current study was to provide proof-of-concept for the proposed carrier phase ultrafiltration and backflow recovery technique. In order to conduct this, model proteins and their mixtures were used. The next step in the development of this technique would be the demonstration of the suitability of this technique for conducting real-life biological separations involving complex feed material. For instance, this technique could be used, in addition to routine sample preparation such as desalting, buffer exchange and concentration, for laboratory-scale purification of large proteins like IgM and even larger species such as viruses, virus-like particles (VLPs), liposomes, vesicles and nanoparticles. Some of these large modalities are shear-sensitive and are therefore likely to degrade while processing with multi-pass, high-shear-rate-based techniques such as TFF. Here, the proposed technique would provide a gentler alternative, albeit with limited processing capacity compared to TFF. Different forms of pulsed ultrafiltration techniques have been used for studying aspects such as protein–drug interactions [46], membrane transport mechanisms [47] and membrane fouling [48]. The proposed technique could therefore also be adopted or adapted for such investigation-type applications. Asymmetric field flow fractionation (or AFF) is a membrane-based separation technique where permeation drag is utilized to separate macromolecules and particles within a flow channel [49]. The application area of the proposed carrier phase ultrafiltration and backflow recovery technique overlaps with that of AFF, and hence separation performance of these techniques will be systematically compared in future studies. In the current study, the sample injection volume and flow rate were not really challenged to any significant extent, as the primary objective was to obtain a proof-of-concept for the proposed technique. If these could be increased, the processing capacity and hence the attractiveness of the technique could be further enhanced. Also, the current study examined single-cycle separations. The low membrane fouling resulting from this technique clearly suggests that it could easily be used for sequential multiple-cycle separations. This aspect will also need to be systematically studied to make the proposed technique suitable for handling even larger volumes of biological material. The technique, as discussed in this paper, uses an existing liquid chromatography system. In a laboratory lacking this facility, a simple chromatography-like system could easily be assembled using inexpensive pumps, detectors and valves. Also, the technique, as discussed in this paper, uses a custom-designed membrane module. Any commercially available TFF membrane module with low hold-up volume could also be used (albeit with some limitations) for carrying out the carrier phase ultrafiltration and backflow recovery technique.

## 4. Conclusions

The results discussed in this paper clearly show that the proposed carrier phase ultrafiltration and backflow recovery technique, that is akin to liquid chromatography, is suitable for medium-to-large volume sample preparation in biological research laboratories. The integration of the membrane module with a liquid chromatography system in a “plug and play” mode made it easy to handle samples and recover products in an unambiguous manner, i.e., with precise input and output volumes. The real-time monitoring of the ultrafiltration and recovery steps using the sensors and detectors of the liquid chromatography system enabled informed decision making about the process, e.g., how long to carry out the diafiltration for or when to recover the enriched product from the membrane module. The concentration of retained species could be enriched near the membrane by utilizing controlled concentration polarization. The use of a pulse of feed in place of a sustained flow of feed resulted in the concentration polarization being self-limiting and hence controllable. The extent of enrichment by controlled concentration polarization could be manipulated by adjusting the volumetric permeate flux. The recovery of retained enriched species without significant dilution was facilitated by backflow of carrier phase. Recovery by advection during backflow was found to be significantly more efficient than recovery by crossflow. The backflow of carrier phase also cleaned the membrane, minimized membrane fouling and readied the membrane module for the next separation cycle. The technique was found to be suitable for carrying out protein fractionation, desalting, buffer exchange and concentration enrichment and is likely to be attractive to non-specialized users of ultrafiltration in biological laboratories. Other attractive features of this technique include high recovery, significant reduction in the number of diavolumes of buffer needed for conducting desalting and buffer exchange and the potential to re-use the membrane module for multiple separation cycles.

## Figures and Tables

**Table 1:** Width at half height of BSA recovery peaks obtained with the different modes of recovery.

Mode of Recovery	Peak Width at Half Height (mL)
Crossflow	7.28
S-backflow	1.35
C-backflow	1.22
Flipped S-backflow	1.41
Flipped C-backflow	1.52

**Table 2:** Overall pressure for filtration of carrier phase (PBS) before and after protein separation experiments.

Type of Experiment	Sample Volume (mL)	Carrier Phase Flow Rate (mL min^−1^)	Pressure before Experiment (MPa)	Pressure after Experiment (MPa)
Lysozyme-BSA fractionation	25	5	0.0703	0.0705
Desalting of 0.25 mg mL^−1^ BSA solution	50	4	0.0647	0.0661
Desalting of 0.25 mg mL^−1^ BSA solution	50	5	0.0713	0.0732
Concentration of 0.25 mg mL^−1^ BSA solution	70	4	0.0643	0.0651
Concentration of 2 mg mL^−1^ BSA solution	50	1	0.0425	0.0432

## Data Availability

The original contributions presented in the study are included in the article, further inquiries can be directed to the corresponding author.
